# Radiation Retinopathy After Whole-Brain Radiotherapy in a Patient With Pineal Gland Tumor

**DOI:** 10.1177/24741264251359075

**Published:** 2025-08-21

**Authors:** Irina Sverdlichenko, Safwan Tayeb, Anarsaikhan Narmandakh, Edward Margolin, Derek S. Tsang, Peng Yan

**Affiliations:** 1 Department of Ophthalmology and Vision Sciences, University of Toronto, Toronto, ON, Canada; 2 Radiation Medicine Program, Princess Margaret Cancer Centre, University Health Network, Toronto, ON, Canada; 3 Donald K. Johnson Eye Institute, University Health Network, Toronto, ON, Canada

**Keywords:** radiotherapy, whole brain, radiation retinopathy

## Abstract

**Purpose:** To describe a case of radiation retinopathy following craniospinal radiation and provide an overview of studies on radiation retinopathy following whole-brain radiotherapy. **Methods:** A retrospective chart review was conducted to collect a single patient’s data on demographics, radiation therapy, ophthalmologic examination findings, treatment, and outcomes. For the systematic review, OVID Medline, Embase, and Cochrane CENTRAL were searched for studies reporting on radiation retinopathy following whole-brain radiotherapy. **Results:** A 24-year-old man with a history of craniospinal radiation presented with proliferative maculopathy. Initial visual acuity was 20/150 in the right eye and 20/40 in the left eye. The patient received monthly intravitreal injections of bevacizumab followed by panretinal laser photocoagulation in both eyes. Maculopathy improved following treatment. Nine articles comprising 13 cases of radiation retinopathy following whole-brain radiotherapy (25 affected eyes; mean ± SD age, 43.3 ± 10.8 years; 46.2% male) were identified by systematic review. None of the patients had diabetes. Radiotherapy was indicated for primary central nervous system lymphoma (6 cases), metastatic spread of breast cancer (4 cases) or lung cancer (2 cases), and acute lymphocytic lymphoma (1 case). The median radiation dose received was 40.7 Gy (range, 30–50). Ten patients had received prior or concurrent chemotherapy. Median time from radiotherapy to symptom onset was 20.5 months (range, 2–216). More than half of patients developed retinopathy bilaterally. **Conclusions:** To our knowledge, this is the first systematic review to highlight the risk of radiation retinopathy following whole-brain radiotherapy, particularly the increased frequency in patients who have received chemotherapy.

## Introduction

Radiation retinopathy involves microangiopathy of the small retinal vessels secondary to endothelial cell loss and capillary closure.^1^ Clinical manifestations of radiation retinopathy include microaneurysms, macular edema, cotton-wool spots, hard exudates, retinal edema, telangiectasia, and perivascular sheathing, which may appear in varying sequence and latency.^2^ The incidence of radiation retinopathy ranges from 3% to 20% and is largely determined by the source, dose, and type of irradiation used.

Since the 1950s, focal or whole-brain radiation therapy has been indicated for central nervous system or head-and-neck tumors in many patients, as well as for brain metastases given its effectiveness in palliation, widespread availability, and ease of delivery.^3^ Radiation retinopathy is seemingly a rare complication of whole-brain radiation. According to a retrospective analysis by Grimm et al,^4^ radiation retinopathy was observed in 18.5% of 5-year survivors after they had received a cumulative radiation dose of 40 to 60 Gy. There are certain risk factors that increase the incidence of radiation retinopathy, including the dose of radiation received by the retina, fractionation schedule, and administration of chemotherapy, as well as patient factors such as presence of systemic illness, primarily diabetes. The aim of this study was to describe a single case of radiation retinopathy and perform a case-based systematic review of all cases of radiation retinopathy that developed after whole-brain radiotherapy.

## Methods

We conducted a retrospective chart review of 1 patient with radiation retinopathy developing after whole-brain radiation. We recorded data on the patient’s age, sex, type of radiation done, and time interval between radiation therapy and onset of radiation retinopathy. We noted the patient’s ophthalmologic examination findings, including dilated fundus examination, optical coherence tomography (OCT), and best-corrected visual acuity (BCVA), as well as treatments and outcomes. Institutional research ethics board approval was received from the University of Toronto. Informed consent was obtained from the patient for use of their clinical data. This study adheres to the tenets of the Declaration of Helsinki.

### Case-Based Systematic Review

We performed a case-based systematic review in accordance with the Cochrane Handbook for Systematic Reviews of Interventions. Reported findings followed the Preferred Reporting Items for Systematic Reviews and Meta-Analyses (PRISMA) guidelines.

Eligibility criteria for the systematic review required that articles be published in English and that the articles report on patients who developed radiation retinopathy following whole-brain radiotherapy. The reviewed outcomes were each patient’s final visual acuity and retina findings. Studies were excluded if radiotherapy was done to treat choroidal melanoma or other orbital tumors. We excluded meta-analyses, reviews, conference papers/abstracts, and nonpublished literature.

For study selection, we searched for articles on OVID Medline, Embase, and Cochrane CENTRAL from the time of study inception to May 2024 (see Tables 1–3 contain details on the search strategies). Grey literature searches and manual searches of references in the original studies and relevant reviews were also conducted. After de-duplication, title and abstract screening was performed, followed by full-text screening.

**Table 1.** Included Cases of Radiation Retinopathy and Overview of Whole-Brain Radiation Treatment From Systematic Review.

Author, Year^ref^	Total (N)	Affected Eyes (n)	Mean Age (Y)	M:F (n)	Other Systemic Diseases (n)	Chemotherapy Prior to Visual Symptoms (n)	Indication for Radiation	Median Total Radiation Dose (Gy)^a^	Fractions (n, duration)^b^	Boost Radiation (Gy)
Grimm, 2006^4^	5	10	40.8 (range, 26–49)	4:1	0	5	Primary CNS lymphoma	43.6	NA	18 Gy to right cerebellum (n = 1); 9 Gy to bilateral frontal lobes (n = 1)
Chan, 2021^5^	1	2	36	1:0	0	0	Brain metastases for primary lung adenocarcinoma	30	10 fractions, over 12 days	NA
Hsu, 2016^6^	1	1	55	0:1	0	0	Brain metastases from primary non-small cell lung cancer	40	16 fractions	NA
Gulati, 2019^7^	1	2	58	1:0	0	1	Primary CNS lymphoma	50	25 fractions	NA
Wiznia, 1994^8^	1	2	18	0:1	0	1	Acute lymphoid leukemia	600 MeV	NA	NA
Sabaner, 2022^9^	1	2	43	0:1	0	1	Metastatic breast cancer	30	10 fractions	NA
Lin, 2014^10^	1	2	56	0:1	0	0	Brain metastases for breast cancer	30	10 fractions	NA
Hurtikova, 2016^11^	1	2	55	0:1	0	1	Brain metastases for breast cancer	30	10 fractions	NA
Ko, 2010^12^	1	2	38	0:1	0	1	Brain metastases for brain cancer	36	NA	NA

Abbreviations: M, male; F, female; CNS, central nervous system; NA, not available.

a Total radiation dose: mean 38.7 Gy, median 40.7 Gy (range, 30–50).

b Radiation fractions: mean 15 fractions, median 10 fractions (range, 10–25).

**Table 2.** Initial Ophthalmologic Examination Findings in the Reviewed Cases.

			Initial LogMAR Visual Acuity in Affected Eye^b^				
Author, Year^Ref^	Median Duration Between Radiation and Visual Symptoms (Months)^a^	Bilateral Involvement (Y/N)	OD	OS	Fundus Examination Findings	OCT Findings	Visual Field Defects
Grimm, 2006^4^	27 (range, 23–216)	Y (n = 5)	NA	NA	Retinal hemorrhages (n = 2), vasculopathy (n = 2), microaneurysms (n = 3)	NA	NA
Chan, 2021^5^	16	Y	0	0	Cotton-wool spots, exudates, dot-blot hemorrhages, with damage concentrated to superior retina	NA	Inferior visual field defects OU
Hsu, 2016^6^	2	N	1.9	NA	Foveal avascular zone with surrounding microaneurysms, telangiectatic vessels	Macular atrophy with diminished photoreceptor IS/OS junction and intraretinal cysts	Paracentral scotoma OD
Gulati, 2019^7^	12	Y	0.48	0.18	Hard exudates in macula, cystoid macular edema and microaneurysms	Cystoid macular edema OU	NA
Wiznia, 1994^8^	2	Y	1.9	1.9	Capillary closure and nonperfusion, bilateral optic disc neovascularization; later developed tractional retinal detachment OU	NA	NA
Sabaner, 2022^9^	NA	Y	1	0.9	Cystoid macular edema OU, serous macular detachment OD	Cystoid macular edema, microaneurysms, capillary drop-out macula	NA
Lin, 2014^10^	18	Y	0.477	0	Intraretinal hemorrhages, cotton-wool spots OU	Macular edema OD	NA
Hurtikova, 2016^11^	72	Y	0.3	0.4	Hard exudates, hemorrhages, macular edema OU	NA	NA
Ko, 2010^12^	8	Y	0.9	1.09	Macular edema, cotton-wool spots, intraretinal hemorrhages OU	Cystic retinal thickening in both maculae	NA

Abbreviations: Y/N, yes/no; OCT, optical coherence tomography; NA, not available.

a Duration between radiotherapy and symptoms: mean, 37.6 months; median, 20.5 months (range, 2–216).

b Initial logMAR visual acuity in affected eye: mean, 0.76; median, 0.477 (range, 0–1.9).

**Table 3.** Treatment and Final Outcomes in the Reviewed Cases.

Author, Year^Ref^	Treatment	Protocol	Median Duration of Follow-up After Retinopathy Diagnosis (Months)^a^	Final LogMAR Visual Acuity in Affected Eye, OD^b^	Vision Change After Treatment, OD	Final LogMAR Visual Acuity in Affected Eye, OS^b^	Vision Change After Treatment, OS	Specific Outcomes/Comments
Grimm, 2006^4^	Laser therapy (n = 2), intraocular steroid injection (n = 2)	NA	114 (range, 10–144)	NA	NA	NA	NA	Functional vision unchanged overall
Chan, 2021^5^	NA	NA	6	NA	NA	NA	NA	No change in vision at follow-up
Hsu, 2016^6^	NA	NA	6	NA	NA	NA	NA	No change in vision at follow-up
Gulati, 2019^7^	Intravitreal bevacizumab, then dexamethasone implant	Bevacizumab 12 doses OD and 3 doses OS over 4 years; then dexamethasone 4 doses OD over 2 years	84	0.48	Unchanged	0.18	Unchanged	Disappearance of hard exudates with reduction of macular edema and microaneurysms
Wiznia, 1994^8^	Panretinal photocoagulation OU	NA	12	1.3	Improved	1.7	Improved	Patient had tractional retinal detachment OU, did not undergo vitrectomy
Sabaner, 2022^9^	Ranibizumab, laser photocoagulation, intravitreal dexamethasone implant	4 injections ranibizumab	NA	0.6	Improved	0.6	Improved	Normal foveal contour and no macular edema
Lin, 2014^10^	Intravitreal Ranibizumab OD	8 injections ranibizumab over 13 months	13	0.22	Improved	0	Unchanged	NA
Hurtikova, 2016^11^	NA	NA	NA	NA	NA	NA	NA	NA
Ko, 2010^12^	IV furosemide for pleural effusion	NA	0.25	0.7	Improved	1.09	Unchanged	Reduction in subfoveal thickness

Abbreviations: NA, not available; IV, intravenous.

a Duration of follow-up after retinopathy diagnosis: mean, 51.7 months; median, 79.2 months (range, 6–84).

b Final visual acuity OU in logMAR: mean 0.64, median 0.54 (range 0–1.09).

Data from each study were extracted in accordance with the PRISMA guidelines. Extracted data included study characteristics, patient demographics, indication for radiotherapy, total radiation dose, duration between radiotherapy and visual symptoms, ophthalmologic examination findings, treatment, and final visual outcomes. The primary measure of interest was the total radiation dose received by the patient; secondary measures of interest were potential risk factors of retinopathy development, including concomitant systemic disease and chemotherapy treatment. Because all eligible studies were case reports and case series, the risk of selection bias was not assessed.

Continuous data from all eligible studies are expressed as the mean ± SD (range). Categorical parameters are shown as a percentage of the total sample.

## Results

### Case Report

**Figure 1.**
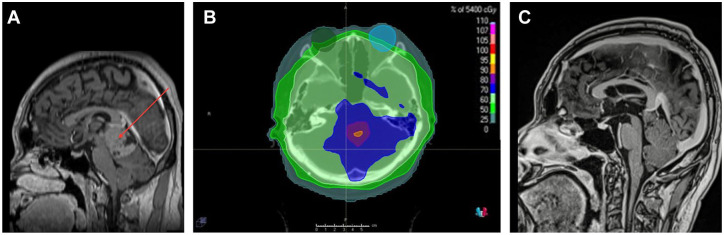
(A) Contrast-enhanced sagittal magnetic resonance imaging (MRI) reconstruction shows a pineal tumor at the time of radiation planning, prior to treatment. (B) Craniospinal irradiation. The right and left globes are shown with green and blue colorwash, respectively. Radiation doses are delineated with colors as shown in the legend. The composite mean radiation doses that the right and left globe received following radiation were 25.3 Gy and 26.7 Gy, respectively. The point maximum radiation doses were 36.7 Gy for both globes. (C) Contrast-enhanced sagittal MRI shows near complete tumor response 2 years postradiation.

A 24-year-old man presented to our ophthalmologic clinic following a 1-month history of blurred vision in both eyes. His medical history noted a pineal parenchymal tumor of World Health Organization severity grade 3 for which he had received photon craniospinal radiation at a dose of 36 Gy (delivered using 5-beam intensity modulated radiation therapy), including radiation of the whole brain. Thereafter, at 1 year prior to presentation to the ophthalmologic service, the patient received focal sequential volumetric modulated arc therapy using conedown boosts to total doses of 54 Gy and 59.4 Gy (Figure 1, A–C). The composite mean radiation doses administered to the right and left globe were 25.3 Gy and 26.7 Gy, respectively. The point maximum radiation doses were 36.7 Gy for both globes. No concurrent chemotherapy was given. At the time of his first ophthalmologic assessment, no optic disc edema was present. No other systemic illnesses, including diabetes or hypertension, were present.

**Figure 2.**
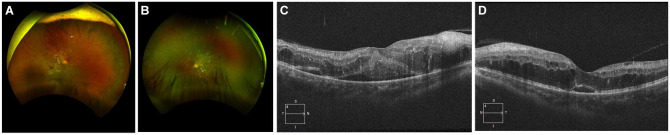
(A) Fundus images of the patient’s right eye at baseline show intraretinal hemorrhages and cotton-wool spots. (B) Fundus image of the patient’s left eye at baseline show intraretinal hemorrhages and cotton-wool spots. (C) Optical coherence tomography (OCT) images of the right eye with cystoid macular edema at baseline. (D) OCT images of the left eye with cystoid macular edema at baseline.

At the initial visit to our clinic, the patient’s tumor was observed to be controlled postradiation, and he had no evidence of malignant disease. His best-corrected visual acuity (BCVA, in Snellen) was 20/150 OD and 20/40 OS. Pupillary examination was normal, with no evidence of a relatively afferent pupillary defect. His color vision, measured by Ishihara plates, was 0/12 OD and 7/14 OS. A dilated fundus examination showed numerous intraretinal hemorrhages, extensive peripapillary cotton-wool spots, and macular edema in both eyes (Figure 2, A–D). There were no signs of papilledema during the initial visit.

**Figure 3.**
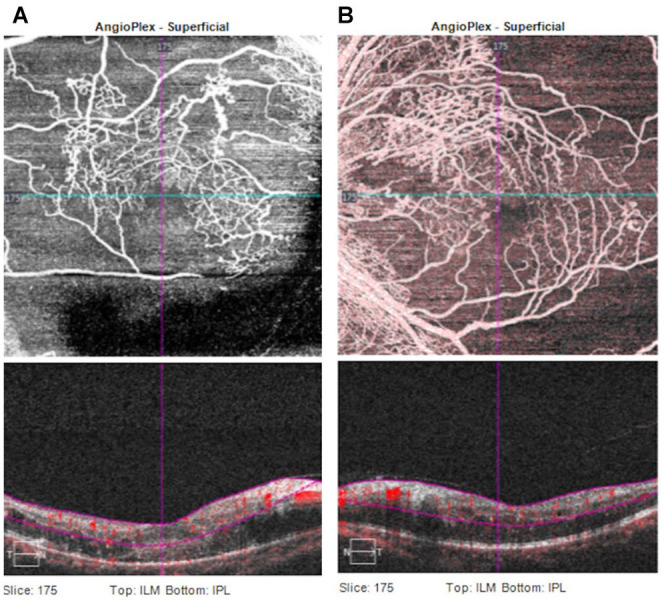
(A) Optical coherence tomography (OCT) angiography images of the right eye show macular ischemia and areas of vascular leakage. (B) OCT angiography images of the left eye show macular ischemia and areas of vascular leakage.

The patient was subsequently assessed by OCT angiography, which demonstrated vascular leakage and marked macular ischemia in both eyes (Figure 3, A and B). The patient was then urgently referred to retina specialists. Given his severe presentation and bilateral signs as well as absence of other systemic illnesses, the specialists deemed the most likely cause of his symptoms to be radiation maculopathy.

**Figure 4.**
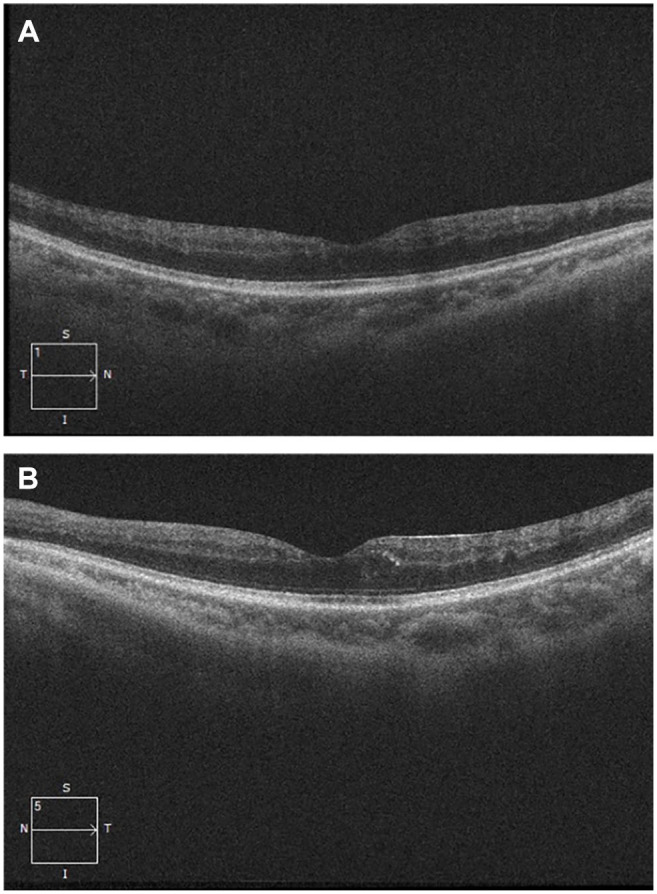
(A) Optical coherence tomography (OCT) images of the right eye taken 2 years after initial presentation. No macular edema is present. (B) OCT images of the left eye taken 2 years after initial presentation. No macular edema is present.

The patient was treated with bevacizumab, receiving a single bevacizumab injection once per month for 5 months in the right eye and once per month for 2 months in the left eye, and was then given panretinal laser photocoagulation therapy in both eyes. At his last follow-up, the maculopathy showed improvement, and no further macular edema was evident (Figure 4). His final Snellen visual acuity was 20/40 OD and 20/60 OS.

### Case-Based Systematic Review

We reviewed the full text of 24 articles to determine eligibility (Supplemental Figure 1). Overall, 9 citations^4–12^ met the full eligibility criteria and were included in the systematic review. Individual study designs and findings are summarized in Tables 1–3.

In the 9 articles eligible for review, we identified a total of 13 cases, including 25 affected eyes, in which radiation retinopathy developed following whole-brain radiotherapy. The mean age of patients in the review sample at the time of presentation was 43.3 ± 10.8 years, and 46.2% (6 of 13) were male. None of the patients had diabetes. The most common indications for radiotherapy were primary central nervous system lymphoma (6 cases), followed by metastatic spread of breast cancer (4 cases), metastatic spread of lung cancer (2 cases), and acute lymphocytic lymphoma (1 case). The median total radiation dose was 40.7 Gy (range, 30–50) over a median of 10 fractions (range, 10–25). Two cases also received boost radiation doses of 18 Gy and 9 Gy, respectively. Information on the dose administered to the globe or retina was not available for any of the cases. Among the 13 patients, 77% (10 of 13) also received chemotherapy prior to developing visual symptoms.

The median time between radiotherapy and onset of visual symptoms was 20.5 months (range, 2–216). Nearly all cases (12 of 13) presented with radiation retinopathy bilaterally on ophthalmologic examination. Findings on fundus examination included retinal hemorrhages (6 cases), microaneurysms (6 cases), cotton-wool spots (4 cases), exudates (3 cases), retinal/macular edema (4 cases), neovascularization (1 case), and serous retinal detachment (1 case). Visual acuity of the affected eyes on the initial ophthalmologic examination was a mean logMAR of 0.76 ± 0.69. Treatments were reported for 7 patients and included intraocular steroid injection (4 cases), intravitreal bevacizumab (1 case), intravitreal ranibizumab (2 cases), and aspirin (1 case). The mean duration of follow-up was 51 months (range, 1 week to 79 months). The final visual acuity was a mean logMAR of 0.64 ± 0.57.

## Conclusions

This case report and systematic review evaluated the presentation and treatment of patients who developed radiation retinopathy following whole-brain radiotherapy. The single case reported herein was of a young male patient with a pineal parenchymal tumor who received craniospinal radiation and subsequently developed radiation retinopathy bilaterally. He was treated with bevacizumab and laser photocoagulation, and showed improvement in his BCVA. The qualitative case-based systematic review assessed the presentation and treatment of 13 patients who received whole-brain radiation for indications other than orbital tumors and developed radiation retinopathy. Nearly half of the patients were male, and none had a concurrent systemic disease. In the 77% of patients who also received chemotherapy prior to developing visual symptoms, the median radiation dose was 41 Gy. The median time from radiation to visual symptom development was 21 months. The treatment type was reported for only 7 patients, including intraocular steroid injection and antivascular endothelial growth factor (anti-VEGF) injections.

Whole-brain radiotherapy is the mainstay treatment administered to patients with identifiable metastases, gross tumor in the head-and-neck region, or primary central nervous system lymphomas. Although rare, radiation retinopathy is a potential complication following whole-brain radiation. It is hypothesized that exposure to radiation causes loss of vascular endothelial cells through direct damage to DNA base pairs, affecting the cells’ ability to divide and causing senescence.^1^ Radiation can also cause indirect damage to cells by exposing the endothelial cells to high concentrations of free radicals, resulting in cell membrane damage. This leads to occlusion of capillary beds and subsequent microaneurysm formation. The clinical features of radiation retinopathy closely resemble diabetic retinopathy, and can include microaneurysms, macular edema, cotton-wool spots, hard exudates, telangiectasia, and perivascular sheathing.

There are several extrinsic and intrinsic factors that increase the risk of radiation retinopathy. The location of the tumor relative to the orbit affects the degree of radiation the retina receives. For example, Monroe et al investigated the incidence of radiation retinopathy in patients receiving radiotherapy for primary tumors of the nasopharynx, paranasal sinuses, and nasal cavity.^13^ They identified a 2% incidence of radiation retinopathy in patients with nasopharyngeal cancer, compared with a 19% incidence in those with nasal cavity tumors and 23% incidence in those with paranasal sinus cancer.

Furthermore, the total cumulative dose of radiation plays an important role in radiation retinopathy formation. Historically, a radiation dose of 45 Gy has been considered a safe threshold.^14^ Monroe et al^13^ reported that 5% of patients (5/103) who received retinal doses of less than 60 Gy developed radiation retinopathy (with a 4% incidence at a dose of <50 Gy), compared with 30% of patients (25/83) who received retinal doses of greater than 60 Gy developing radiation retinopathy; these higher retinal doses (>60 Gy) were significantly associated with its development. Similarly, Parsons et al^15^ noted a dramatic increase in incidence of radiation retinopathy at retinal doses between 45 Gy and 55 Gy, such that nearly all patients receiving higher doses developed retinopathy. That being said, risk evaluations have suggested that the radiation safety threshold should be lower, at doses ranging between 30 and 35 Gy.^16–18^ The total median dose of 41 Gy administered to patients in the studies from our systematic review demonstrates that radiation retinopathy can still occur at lower radiation doses. Moreover, in our presented case, exposure of the globe and retina to doses of less than 37 Gy led to retinopathy in both eyes.

Finally, the fractionation schedule can affect the development of retinopathy. Previous studies have shown that administration of less than 1.9 Gy per fraction can decrease the incidence of retinopathy.^13^ However, the fractionation schedule in our systematic review ranged from 2 Gy per fraction to 3 Gy per fraction, which could increase the risk of retinopathy. Many of these patients were being treated for brain metastases, and thus the large radiation dose selected could have been palliative. The average fractionation schedule may differ in patients being treated for primary disease, thus influencing the risk of downstream radiation retinopathy development.

Another important external factor is use of prior or concomitant chemotherapy. Use of chemotherapy, either prior to or concurrent with radiation, is thought to make the retinal vasculature more vulnerable to radiation damage by increasing oxygen-derived free radicals.^19^ Interestingly, nearly 80% of the patients in our systematic review received chemotherapy before their visual symptoms developed. In these cases, the chemotherapy may have augmented the retinal vasculature damage caused by whole-brain radiation, thus increasing the likelihood of radiation retinopathy development.

Finally, one of the most important intrinsic risk factors for radiation retinopathy is the concurrent presence of diabetes. There appears to be a synergistic action of radiation and diabetes on the capillaries that predisposes the eyes to retinopathy.^20^ The cumulative effect of pericyte damage seen in patients with diabetes and endothelial damage seen in those exposed to radiation causes severe occlusive arteritis, which is commonly seen in radiation retinopathy. Diabetes has also been associated with poor visual outcomes due to higher incidence of neovascular glaucoma and diabetic papillopathy.^21^ None of the articles identified in our systematic review described a patient with diabetes.

Only a few studies in our review reported on therapies for radiation retinopathy, and therefore it is difficult to comment on specific treatment outcomes. In general, treatments can include anti-VEGF, intraocular steroid injection, and laser photocoagulation. VEGF and other inflammatory and vasculogenic factors have been implicated in the pathogenesis of radiation-induced macular edema and neovascularization. Thus, many clinicians have opted to use anti-VEGF medications to treat radiation macular edema.^22^ Finger et al^23^ demonstrated that continuous injections of anti-VEGF in 4-week to 12-week intervals led to progressive reductions in macular edema, exudates, and cotton-wool spots. Additionally, the probability of remaining within 2 lines of initial visual acuity was 69% at 5 years and 38% at 8 years of anti-VEGF therapy.^23^

Intravitreal steroids are thought to stabilize endothelial tight junctions, prevent leukocyte migration, and inhibit synthesis of prostaglandins, proinflammatory cytokines, and VEGF.^24^ Shields et al^24^ studied intravitreal triamcinolone acetonide as a treatment for radiation maculopathy and found that while 91% of patients experienced vision stabilization or improvement at 1 month of treatment, only 45% retained stable visual acuity at 6 months. Furthermore, laser photocoagulation therapy decreases the leakage from abnormally permeable retinal vessels, reduces the proliferation of new retinal vessels, and limits the neovascularization stimulus by converting hypoxic retina into an anoxic state.^25^

This study has several strengths. To our knowledge, this is the first systematic review to describe cases of radiation retinopathy that developed following whole-brain radiotherapy. It provides a comprehensive overview of patient factors, ophthalmologic findings, treatments, including radiation dosimetry, and outcomes in these patients, thus contextualizing the need for close monitoring for development of retinopathy following brain radiotherapy. However, we acknowledge certain limitations of the study. The systematic review contained only case reports and case series, which represent the lowest level of evidence and contain inherent bias. Due to the nature of the study designs in the reviewed articles and the fact that there were only 13 cases, we are not able to predict causality with regard to the risk factors for development of radiation retinopathy, and we cannot characterize the treatment outcomes with different therapies. The exact dose of radiation administered to the retina or globe was available for the presented case, but not for cases from the historical literature. Finally, almost all patients in the systematic review received prior or concurrent chemotherapy, which is a known risk factor for radiation retinopathy. It would be interesting to conduct a prospective study on the incidence of radiation retinopathy following whole-brain radiotherapy in patients who receive chemotherapy in comparison to those who do not receive chemotherapy.

This case report and systematic review of radiation retinopathy following whole-brain radiotherapy shows that, although radiation retinopathy is a rare complication following brain radiotherapy, it can still occur at whole-brain radiation doses of less than 45 Gy. While no patients had any systemic illnesses, most patients who developed radiation retinopathy received chemotherapy prior to visual symptom development. The treatments for retinopathy described in the reviewed studies included anti-VEGF and intraocular steroids. Clinicians need to recognize the potential complication of retinopathy following brain radiotherapy, particularly the increased risk following chemotherapy. Regular ophthalmologic investigations should be performed to ensure early diagnosis and treatment for these patients.

## Supplemental Material

sj-docx-1-vrd-10.1177_24741264251359075 – Supplemental material for Radiation Retinopathy After Whole-Brain Radiotherapy in a Patient With Pineal Gland Tumor Supplemental material, sj-docx-1-vrd-10.1177_24741264251359075 for Radiation Retinopathy After Whole-Brain Radiotherapy in a Patient With Pineal Gland Tumor by Irina Sverdlichenko, Safwan Tayeb, Anarsaikhan Narmandakh, Edward Margolin, Derek S. Tsang and Peng Yan in Journal of VitreoRetinal Diseases

sj-docx-2-vrd-10.1177_24741264251359075 – Supplemental material for Radiation Retinopathy After Whole-Brain Radiotherapy in a Patient With Pineal Gland Tumor Supplemental material, sj-docx-2-vrd-10.1177_24741264251359075 for Radiation Retinopathy After Whole-Brain Radiotherapy in a Patient With Pineal Gland Tumor by Irina Sverdlichenko, Safwan Tayeb, Anarsaikhan Narmandakh, Edward Margolin, Derek S. Tsang and Peng Yan in Journal of VitreoRetinal Diseases

sj-docx-3-vrd-10.1177_24741264251359075 – Supplemental material for Radiation Retinopathy After Whole-Brain Radiotherapy in a Patient With Pineal Gland Tumor Supplemental material, sj-docx-3-vrd-10.1177_24741264251359075 for Radiation Retinopathy After Whole-Brain Radiotherapy in a Patient With Pineal Gland Tumor by Irina Sverdlichenko, Safwan Tayeb, Anarsaikhan Narmandakh, Edward Margolin, Derek S. Tsang and Peng Yan in Journal of VitreoRetinal Diseases

sj-docx-4-vrd-10.1177_24741264251359075 – Supplemental material for Radiation Retinopathy After Whole-Brain Radiotherapy in a Patient With Pineal Gland Tumor Supplemental material, sj-docx-4-vrd-10.1177_24741264251359075 for Radiation Retinopathy After Whole-Brain Radiotherapy in a Patient With Pineal Gland Tumor by Irina Sverdlichenko, Safwan Tayeb, Anarsaikhan Narmandakh, Edward Margolin, Derek S. Tsang and Peng Yan in Journal of VitreoRetinal Diseases
