# Supplementary material for: Radiation Retinopathy After Whole-Brain Radiotherapy in a Patient With Pineal Gland Tumor
Source: J Vitreoretin Dis. 2025 Aug 21;9(5):678–86. doi: 10.1177/24741264251359075 (PMC12370669; doi:10.1177/24741264251359075)
Supplement: Supplementary file 4 [file sj-docx-4-vrd-10.1177_24741264251359075.docx]

| **#** | **Searches** | **Results from February 5, 2023** |
| --- | --- | --- |
| 1 | Title Abstract Keyword: “Retinopathy” | 0 |
| 2 | Title Abstract Keyword: “whole brain radiation therapy” or “whole brain radiotherapy” or “whole-brain radiation therapy” or “whole-brain radiotherapy” or “cranial radiation therapies” or “cranial radiation” |  |

Supplementary Table 3 – COCHRANE CENTRAL Search
