# Supplementary material for: Radiation Retinopathy After Whole-Brain Radiotherapy in a Patient With Pineal Gland Tumor
Source: J Vitreoretin Dis. 2025 Aug 21:24741264251359075. Online ahead of print. doi: 10.1177/24741264251359075 (PMC12370669; doi:10.1177/24741264251359075)
Supplement: sj-docx-1-vrd-10.1177_24741264251359075 – Supplemental material for Radiation Retinopathy After Whole-Brain Radiotherapy in a Patient With Pineal Gland Tumor [file sj-docx-1-vrd-10.1177_24741264251359075.docx]

Supplementary Figure 1: Preferred Reporting Items for Systematic Reviews and Meta-Analyses (PRISMA)

Studies included in qualitative synthesis (n=9)

Full-text articles excluded (n=15)

No full text – 6

No English translation – 3

No whole brain radiotherapy - 5

Post-orbital radiotherapy - 1

Full text articles assessed for eligibility (n=24)

Records excluded (n=117)

Records screened (n=138)

Additional records identified through Cochrane Library, Grey literature, and manual reference search (n=5)

Records identified through OVID Medline/EMBASE search (n=133)
