# Supplementary material for: Radiation Retinopathy After Whole-Brain Radiotherapy in a Patient With Pineal Gland Tumor
Source: J Vitreoretin Dis. 2025 Aug 21:24741264251359075. Online ahead of print. doi: 10.1177/24741264251359075 (PMC12370669; doi:10.1177/24741264251359075)
Supplement: sj-docx-2-vrd-10.1177_24741264251359075 – Supplemental material for Radiation Retinopathy After Whole-Brain Radiotherapy in a Patient With Pineal Gland Tumor [file sj-docx-2-vrd-10.1177_24741264251359075.docx]

Supplementary Table 1 – OVID MEDLINE Search

| **#** | **Query** | **Results from 13 May 2024** |
| --- | --- | --- |
| 1 | Retinopath.tw,kf. | 59073 |
| 2 | ((brain* or cranial* or skull) and (radiation* or irradiat* or radio*)).tw,kf. | 111223 |
| 5 | 2 and 3 | 120 |
| 6 | Limit 7 to humans | 99 |
