# Supplementary material for: Radiation Retinopathy After Whole-Brain Radiotherapy in a Patient With Pineal Gland Tumor
Source: J Vitreoretin Dis. 2025 Aug 21:24741264251359075. Online ahead of print. doi: 10.1177/24741264251359075 (PMC12370669; doi:10.1177/24741264251359075)
Supplement: sj-docx-3-vrd-10.1177_24741264251359075 – Supplemental material for Radiation Retinopathy After Whole-Brain Radiotherapy in a Patient With Pineal Gland Tumor [file sj-docx-3-vrd-10.1177_24741264251359075.docx]

Supplementary Table 2 – OVID EMBASE Search

| **#** | **Query** | **Results from 13 May, 2024** |
| --- | --- | --- |
| 1 | Retinopathy/ | 24758 |
| 2 | Retinopath.*tw,kf. | 86630 |
| 3 | Whole brain radiotherapy/ | 5528 |
| 4 | Skull irradiation/ | 5207 |
| 5 | ((brain* or cranial* or skull*) and (radiation* or radio* or irrad*)).tw.kf. | 179476 |
| 6 | 1 or 2 | 93926 |
| 7 | 3 or 4 or 5 | 182437 |
| 8 | 6 and 7 | 243 |
| 9 | Limit 8 to human | 220 |
